# Animal model contributes to the development of intracranial aneurysm: A bibliometric analysis

**DOI:** 10.3389/fvets.2022.1027453

**Published:** 2022-11-18

**Authors:** Jia Chen, Jing Liu, Xin Liu, Chudai Zeng, Zhou Chen, Shifu Li, Qian Zhang

**Affiliations:** ^1^Xiangya Nursing School, Central South University, Changsha, China; ^2^The Chinese People's Liberation Army 921 Hospital of Joint Logistics Support Force, Department of General Practice, Changsha, China; ^3^Department of Neurosurgery, Xiangya Hospital, Central South University, Changsha, China; ^4^Hypothalamic-Pituitary Research Center, Xiangya Hospital, Central South University, Changsha, China; ^5^National Clinical Research Center for Geriatric Disorders, Xiangya Hospital, Central South University, Changsha, China

**Keywords:** intracranial aneurysms (IAs), animal model, bibliometrics, VOSviewer, CiteSpace, keyword co-occurrence analysis

## Abstract

**Introduction:**

Studies on intracranial aneurysms (IAs) using animal models have evolved for decades. This study aimed to analyze major contributors and trends in IA-related animal research using bibliometric analysis.

**Methods:**

IA-related animal studies were retrieved from the Web of Science database. Microsoft Excel 2010, GraphPad Prism 6, VOSviewer, and CiteSpace were used to collect and analyze the characteristics of this field.

**Results:**

A total of 273 publications were retrieved. All publications were published between 1976 and 2021, and the peak publication year is 2019. Rat model were used in most of the publications, followed by mice and rabbits. Japan (35.5%), the United States (30.0%), and China (20.1%) were the top three most prolific countries. Although China ranks third in the number of publications, it still lacks high-quality articles and influential institutions. *Stroke* was the most prolific journal that accepted publications related to IA research using animal models. *Circulation* has the highest impact factor with IA-related animal studies. Hashimoto N contributed the largest number of articles. Meng hui journal published the first and second highest cited publications. The keywords “subarachnoid hemorrhage,” “macrophage,” “rupture,” “mice,” “elastase,” “gene,” “protein,” “proliferation,” and “risk factors” might be a new trend for studying IA-related animal research.

**Conclusions:**

Japan and the Unites States contributed the most to IA–related animal studies, in terms of both researchers and institutions. Although China ranks third in terms of the number of publications, it should strengthen the quality of its publications. Researchers should pay attention to the latest progress of *Stroke, Journal of Neurosurgery, Neurosurgery*, and *Circulation* for their high-quality IA-related animal studies. Using animal IA models, especially mice, to investigate the molecular mechanisms of IA may be the frontier topic now and in future.

## Introduction

Intracranial aneurysm (IA) is characterized by abnormal dilation of the cerebral artery and can create a life-threatening situation if the artery is ruptured (1). It is a common vascular abnormality with a lifetime prevalence of 3–5%, and a rupture rate of 1% in the general population (2). Various acquired and inherited factors may influence the initiation, progression, and rupture of IA, including age, sex, hypertension, and smoking (3). Apart from conservative treatment, the treatment for IA has shifted from surgical clipping to minimally invasive approaches (e.g., coiling, stenting, and flow diversion) in recent years (4). To date, the exact pathophysiologic mechanism of IA remains elusive, and no effective therapeutic drug has been identified based on the understanding of the molecular and cellular mechanisms underlying aneurysm formation. In addition, a lack of early-stage human aneurysm tissues hampered the understanding of early cellular and molecular changes in aneurysms (5). A reliable animal model, which accurately recapitulates IA pathogenesis *in vivo*, is an important tool for studying the mechanism of IA (6). Currently, several animal IA models are used, including mice, rats, and rabbits (7). However, selecting an appropriate animal IA model may be difficult for new researchers as the selection depends on former publications. Thus, it is important to find the key publications and understand the state-of-the-art techniques in this field. Bibliometric analysis is used to analyze the current status and frontier topics in this field (8–10). Previous studies explored the trend of animal models used for Alzheimer's disease (11) and scoliosis (12). In addition, our previous study suggests a future research direction of studying the pathogenesis of IA from the perspective of inflammation-related genes and hemodynamics (13). Therefore, the present bibliometric analysis aimed to summarize the properties of the IA-related animal studies from the Web of Science (WOS) database. The bibliometric analysis uncovered and visualized the following research questions (RQs):

RQ1. The publication trend for IA research using different animals.

RQ2. The most contributing authors, institutions, countries, journals, and their cooperative networks.

RQ3. The most influential publications in this field.

RQ4. The current and frontier topics for IA research using different animals.

## Methods and materials

### Search strategy

To avoid bias introduced by database retrieval, we performed an electronic search using the Web of Science (WOS). Expanded database (Thomson Reuters, New York, USA) from its date of inception (1945) to 1 July 2021. The Web of Science (WOS, Clarivate Analytics, Philadelphia, PA, USA) is one of the most professional and authoritative citation-based databases with a powerful indexing function, which contains not only basic bibliometric parameters, including title, author, institution, country/region, and author keywords, but also reference information. Thus, WOS has been widely used in bibliometric studies (14, 15).

To obtain publications primarily focused on animal IA models, an advanced search was performed using the following items: TI = (“cerebral aneurysm^*^” or “intracranial aneurysm^*^” or “brain aneurysm^*^”) and TS= (“animal model” or “zebrafish” or “mice” or “mouse” or “murine” or “rat” or “rabbit” or “canine” or “dog” or “pig” or “swine” or “monkey” or “gorilla” or “primate”). First, two investigators (Qian Zhang and Jing Liu) independently searched and screened the database. Second, Shifu Li and Jia Chen re-evaluated the obtained publications by reading the title and abstract. Any discrepancies were resolved by discussion until a consensus was reached.

### Selection criteria

IA studies involving animal models were selected for the final analysis. Publications primarily focusing on subarachnoid hemorrhage (SAH) and clinical outcomes of patients were excluded. Our search was limited to articles published in English.

### Data extraction

The following bibliometric parameters were extracted: title, keywords, authors, journal, year, citation counts, country, institution, and species of animal (e.g., rat or mice). The data were then imported into Microsoft Excel 2010 (Redmond, Washington, USA), GraphPad Prism 6 (GraphPad Prism Software Inc., San Diego, CA), VOSviewer (Leiden University, Leiden, the Netherlands), and CiteSpace (version 5.8 R3); then, the major contributors were analyzed, and the knowledge network was visualized (16, 17).

## Results

### Search results

**Figure 1:**
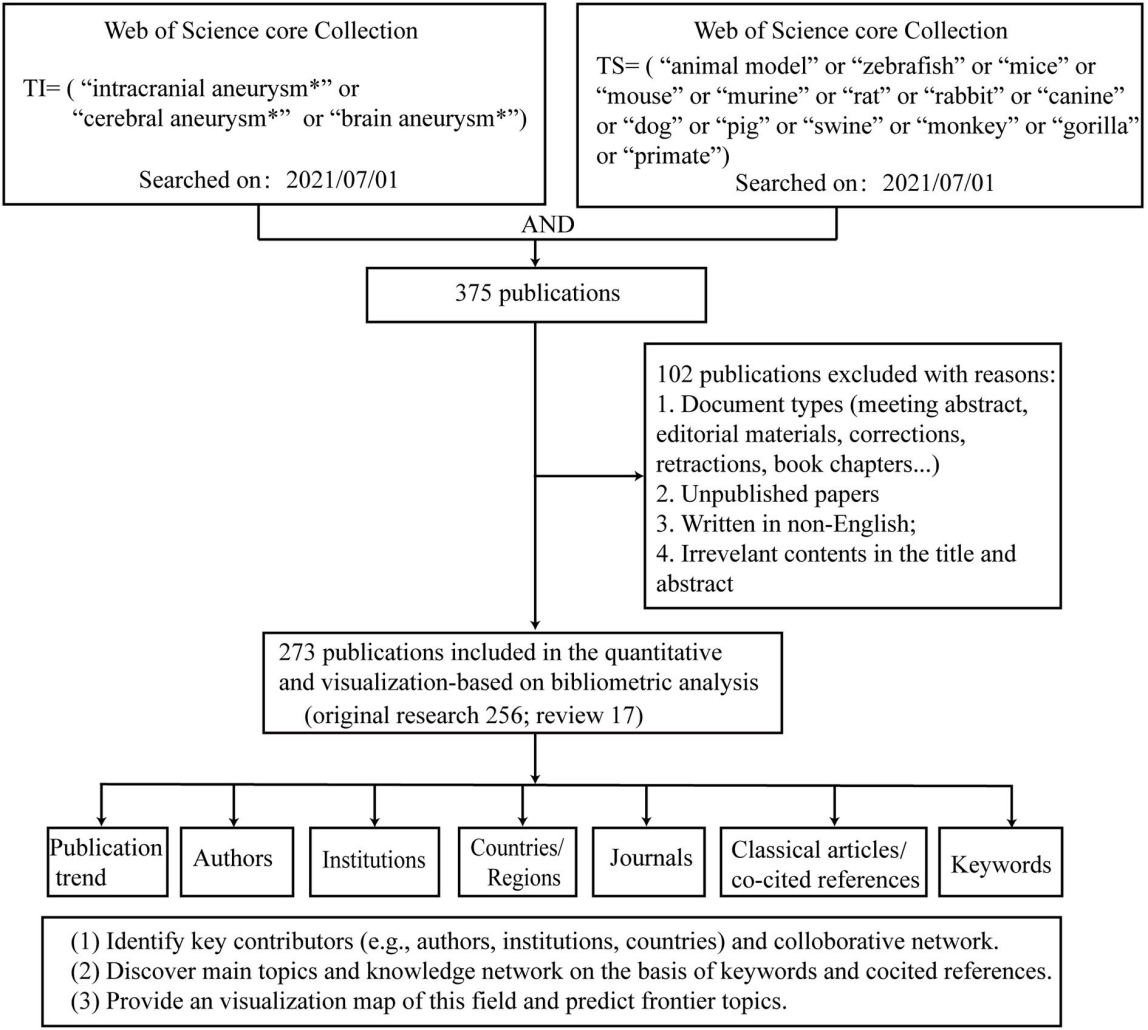
Flowchart of data screening and analyzing.

The initial search of the WOS identified 375 publications. After limiting the search to English language articles and excluding irrelevant publications that focused on SAH and that lacked keywords and cited references (e.g., letters, corrections, and books), 273 publications were obtained. Of the 273 articles, 256 publications were original research and 17 publications were review articles (Figure 1).

### Annual scientific production

**Figure 2:**
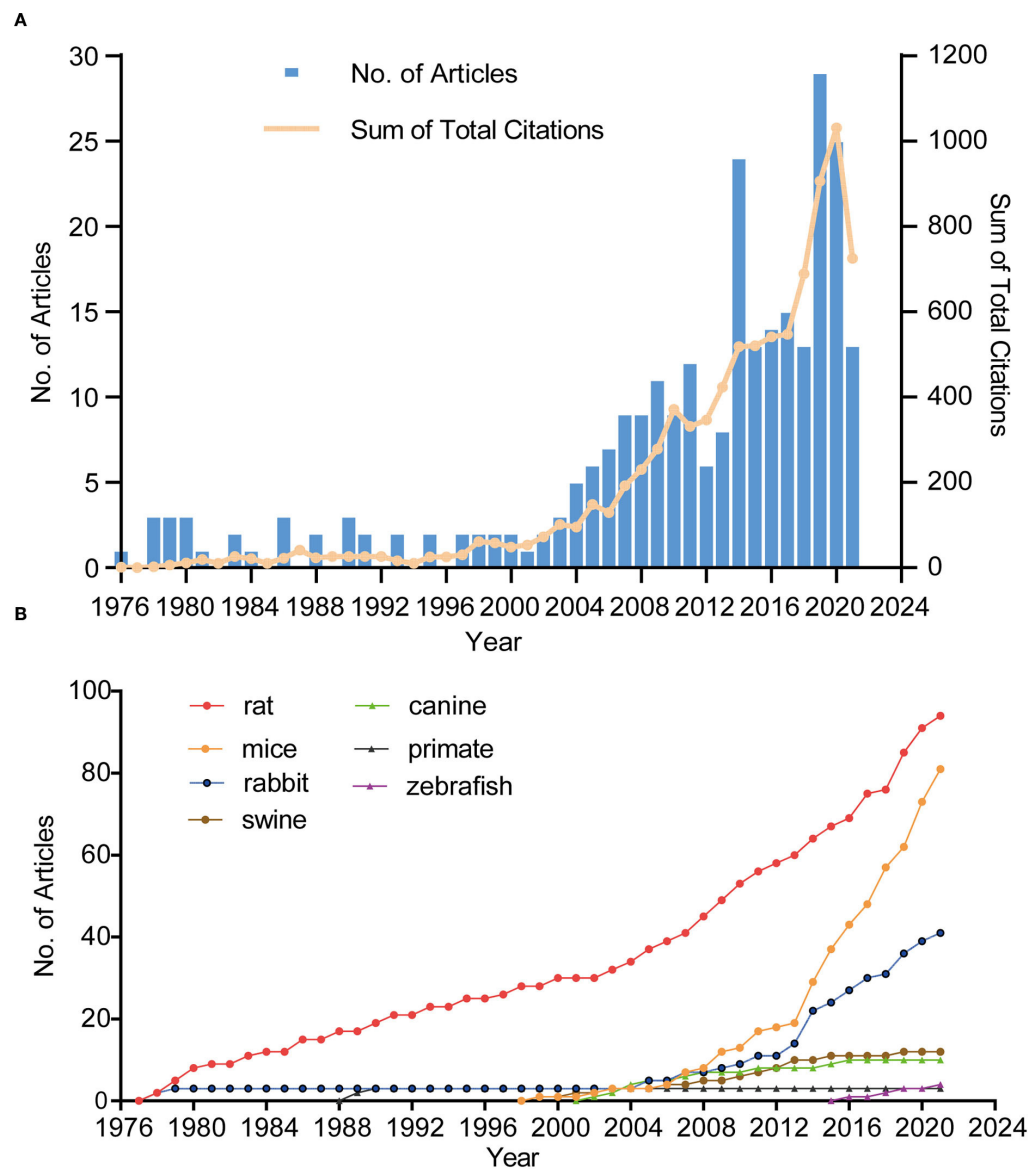
The trend in terms of publications, citations **(A)**, and different species **(B)**. Blue bars represent the number of articles related to animal IA model per year. The yellow line represents the trend of total citations in this field.

Figure 2A shows the annual publications and total citations of IA-related animal research from its first inception (1976) to 2021. The published time can be divided into two phases (phase 1, 1976–2003; phase 2, 2004–2021). In phase 1, the number of publications was <5, whereas, in phase 2, the number of publications increased by 4-fold, from five (2004) to 25 (2021). Of the 10 most cited publications, seven articles were published before 2010 (one in the 1970's, one in the 1980's, and six in the 2000's), and the remaining two articles were published after 2010 (Table 1). In 1976, Santos-Buch et al. published the first study on this topic in the Archives of Neurology, entitled “*Concurrence of iris aneurysms and cerebral hemorrhage in hypertensive rabbits*.” The authors found that a striking rate (42.5%) of hemorrhagic stroke occurred during the formation of iris aneurysms in hypertensive rabbits (18). In 1978, Hashimoto et al. published “*Experimentally induced cerebral aneurysms in rat*” in *Surgical Neurology*. He had successfully induced saccular cerebral aneurysms in rats, which were treated with beta-aminopropionitrile, deoxycorticosterone, and salt hypertension with unilateral common carotid artery ligated (19). This analysis might be the milestone of IA-related animal research (7, 20–23).

**Table 1:** Top 10 most cited publications.

**Rank**	**Title**	**Total citations**	**Citations/year**	**Publication year**	**First author**	**Journal**	**Country**
1	Complex hemodynamics at the apex of an arterial bifurcation induces vascular remodeling resembling cerebral aneurysm initiation	381	25.4	2007	Meng, Hui	Stroke	USA
2	High WSS or Low WSS? Complex Interactions of Hemodynamics with Intracranial Aneurysm Initiation, Growth, and Rupture: Toward a Unifying Hypothesis	368	46	2014	Meng, Hui	American Journal of Neuroradiology	USA
3	Macrophage-derived matrix metalloproteinase-2 and-9 promote the progression of cerebral aneurysms in rats	210	14	2007	Aoki, Tomohiro	Stroke	Japan
4	Critical Roles of Macrophages in the Formation of Intracranial Aneurysm	184	16.73	2011	Kanematsu, Yasuhisa	Stroke	USA
5	Experimentally induced cerebral aneurysms in rats.	183	4.16	1978	Hashimoto, N	Surgical Neurology	Japan
6	Matrix and Bioabsorbable polymeric coils accelerate healing of intracranial aneurysms - Long-term experimental study	181	9.53	2003	Murayama, Y	Stroke	USA
7	Prevention of rat cerebral aneurysm formation by inhibition of nitric oxide synthase	173	7.86	2000	Fukuda, S	Circulation	Japan
8	Intracranial aneurysms: links among inflammation, hemodynamics and vascular remodeling	164	10.25	2006	Hashimoto, Tomoki	Neurological Research	USA
9	NF-kappa B is a key mediator of cerebral aneurysm formation	161	10.73	2007	Aoki, Tomohiro	Circulation	Japan
10	Experimentally induced cerebral aneurysms in rats: Part V. Relation of hemodynamics in the circle of Willis to formation of aneurysms.	159	3.79	1980	Hashimoto, N	Surgical Neurology	Japan

### Analysis of species

In terms of species, rats were used in most of the studies (*n* = 94), followed by mice (*n* = 81) and rabbits (*n* = 41). Approximately 80% of the studies used these three animals. The first publication year of IA research involving rats, mice, rabbits, swine, canines, zebrafish, primates was in 1978 (19), 1999 (24), 1976 (18), 1997 (25), 2002 (26), 2016 (27), and 1987 (28), respectively. Figure 2B shows a steady and slow increase in rat-related studies since the inception of this field but a sharp increment in mouse-related studies since 2006.

### Citations

The median number of citations was 32.37 (range, 0–381), and the number of citations per publication was 0.7 (range, 0–46.25). The 10 most cited publications are listed in Table 1. Of the 10, four publications used rats as the IA model (19, 29–31), two review articles (32, 33), one study using both rats and mice (34) and the remainingusing mice (35), rabbits (36), and swine (37). The highest cited publication (*n* = 381 total citations) was the study by Meng et al. (36), entitled “*Complex hemodynamics at the apex of an arterial bifurcation induces vascular remodeling resembling cerebral aneurysm initiation*” in *Stroke*. In this study, to observe aneurysm-like remodeling, the authors surgically created new branch points in the carotid artery of six female adult dogs after 2 weeks or 2 months. Using computational fluid dynamics simulations and histological detection, the authors found that the combination of elevated wall shear stress and high wall shear stress gradients formed a potentially “dangerous” hemodynamic microenvironment. This hemodynamic microenvironment predisposes a vessel wall at or near bifurcation apices to aneurysm formation (36). Interestingly, the second most cited publication (n=368 citations) was the study by Meng et al., entitled “*High WSS or low WSS? Complex Interactions of Hemodynamics with Intracranial Aneurysm Initiation, Growth, and Rupture: Toward a Unifying Hypothesis*” in American Journal of Neuroradiology, with 46.25 citations per year. They pointed out that the different hemodynamics (e.g., low wall shear stress or high wall shear stress) could crosstalk with different cells, such as inflammable cells or vascular mural cells, to mediate the initiation, progression, and rupture of IA (32).

### Authors

A total of 1022 scholars were involved in the selected IA-related animal studies. Table 2 lists the 10 most prolific authors. Of the 10 most productive authors, eight authors were from Japan and two were from the United States. Among the Japanese authors, six were from the Kyoto University Graduate School of Medicine and two were from Tokushima University Graduate School. Hashimoto N (Kyoto University Graduate School of Medicine, Japan) ranked first, with 44 articles and 2,861 citations, followed by Aoki T (Kyoto University Graduate School of Medicine, Japan), with 42 articles and 1,726 total citations.

**Table 2:** Top 10 prolific authors.

**Author**	**Country**	**Affiliation**	**Publications**	**TC**
Hashimoto N	Japan	Department of Neurosurgery, Kyoto University, Graduate School of Medicine	44	2861
Aoki T	Japan	Innovation Center for Immunoregulation Technologies and Drugs (AK project), Kyoto University Graduate School	42	1726
Kataoka H	Japan	Department of Neurosurgery, Kyoto University, Graduate School of Medicine	34	1596
Nozaki K	Japan	Department of Neurosurgery, Kyoto University, Graduate School of Medicine	32	1709
Hashimoto T	USA	Departments of Neurosurgery and Neurobiology, Barrow Aneurysm and AVM Research Center, Barrow Neurological	20	865
Hazama F	Japan	Department of Neurosurgery, Kyoto University Medical School and Hospital	19	1106
Kitazato KT	Japan	Department of Neurosurgery, Institute of Biomedical Sciences, Tokushima University Graduate School	19	820
Ishibashi R	Japan	Department of Neurosurgery, Kyoto University Graduate School of Medicine	17	999
Nagahiro S	Japan	Department of Neurosurgery, Institute of Biomedical Sciences, Tokushima University Graduate School	17	758
Tada Y	USA	Department of Anesthesia and Perioperative Care, University of California	17	722

TC, total citations.

### Institutions

**Figure 3:**
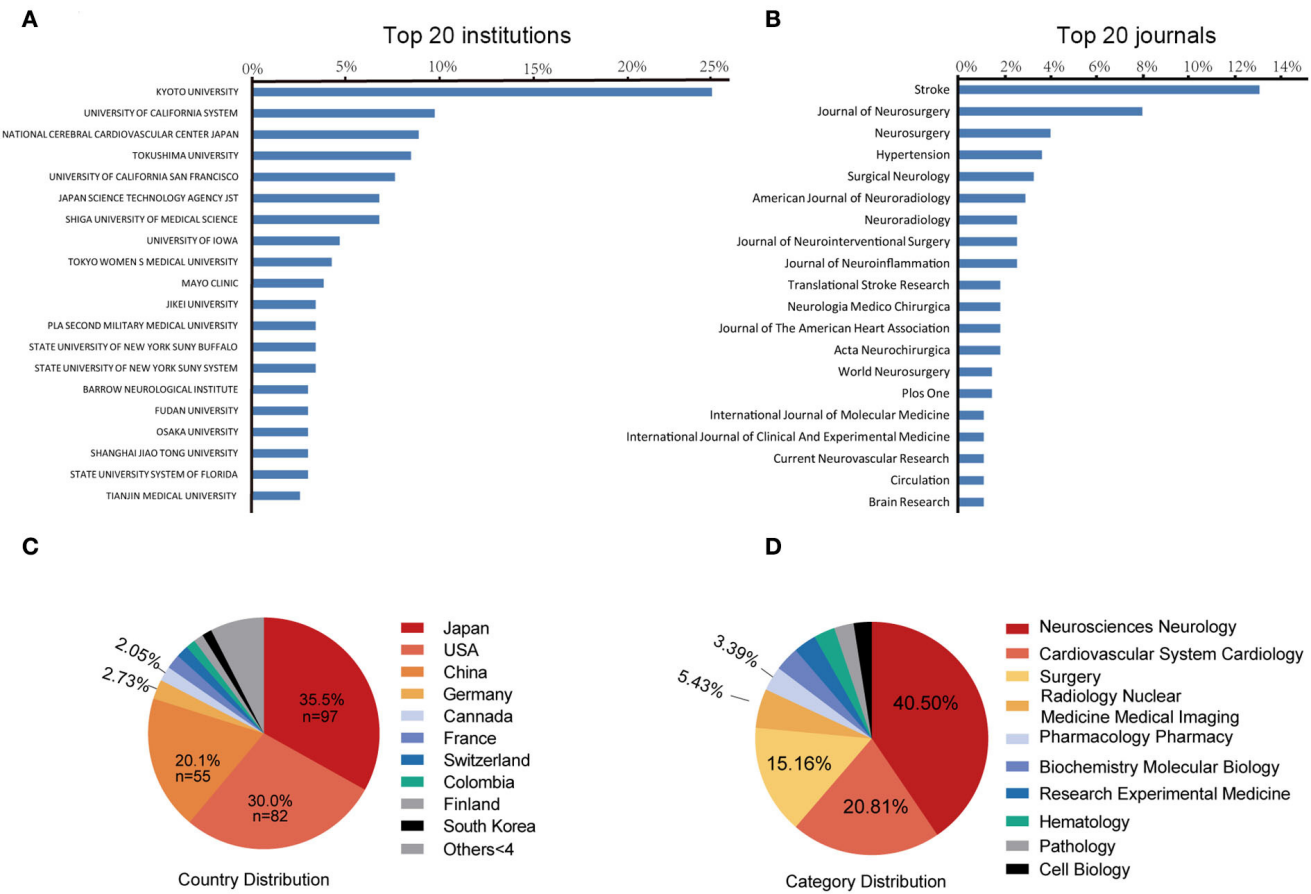
The top 20 most prolific institutions **(A)** and journals **(B)**. Blue bars indicate the proportion of the total number of publications. The 10 most prolific countries **(C)** and research category **(D)**. The area of each country is proportional to the total number of publications.

A total of 301 institutions participated in IA-related animal studies. The Kyoto University Graduate School of Medicine (Japan) ranked first, with 58 publications (24.37% of the total), followed by the University of California (USA), with 23 publications, and the National Cerebral Cardiovascular Center (Japan), with 21 publications. Figure 3A lists the top 20 most prolific institutions, which include eight Japanese institutions, eight U.S. institutions, and four Chinese institutions.

### Journals

A total of 104 journals accepted IA-related animal studies. Figure 3B lists the top 20 most prolific journals. The most productive journal is *Stroke* (IF = 10.17, 2021), with 36 publications (13.1% of the total), followed by the *Journal of Neurosurgery* (IF = 5.41, 2021), with 22 publications (8.0%), and *Neurosurgery* (IF = 5.31, 2021), with 11 publications (4.0%). Of the ten most cited publications, four articles were published in *Stroke*. *Circulation* has the highest impact factor (IF = 39.92, 2021) in this field.

### Countries

**Figure 4:**
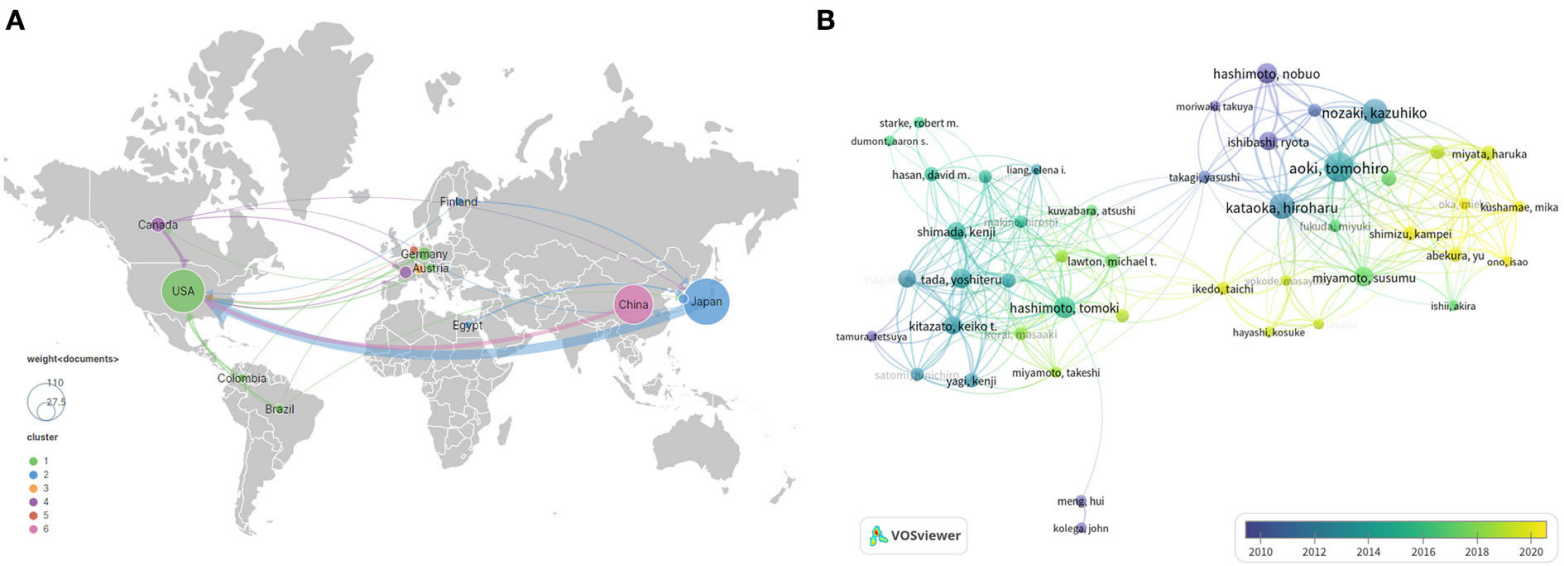
International cooperation **(A)** and author cooperation network **(B)** identified by VOSviewer. Node size indicates the number of articles produced. The width of links positively associates with cooperation strength. The node color reflects the corresponding average appearing year according to the color gradient in the lower right corner, and dark blue or light blue represents studies that appeared relatively earlier, whereas green yellow or yellow represents studies active in recent years.

A total of 35 countries have published IA-related animal studies. Figure 3C lists the prolific countries that published more than five articles in this field. Japan ranked first, with 97 publications (35.5% of the total), followed by the United States, with 82 publications (30.0%), and China, with 55 publications (20.1%). Country co-authorship maps in VOSviewer were used to understand existing international collaborations. We analyzed the country-wise distribution in this field using VOSviewer, and the results were exported into SCimago Graphica (https://graphica.app/). Countries in one cluster with the same color were closely cooperated (16). The more the number of studies published in a country, the larger the circle; the stronger the collaboration between two countries, the thicker the connecting line (17). Figure 3D shows the category distribution of IA animal model field. The top three categories are Neurosciences neurology, Cardiovascular system cardiology, and Surgery. They accounted 76% publications in this field. As shown in Figure 4A, Japan, the United States, and China are the central nodes in the international cooperation network, among which the United States cooperated the most with other countries, with a total link strength (TLS) of 48, followed by Japan, with a TLS of 28, and China, with a TLS of 9. China and Japan have the most cooperation with the United States. To display the active period of scholars in this field, we used the timeline view in VOSviewer, where color denotes the average publication year, suggesting its active time. Blue indicates relatively early time, while yellow means more recently (38, 39). In Figure 4B, prolific authors who published more than five articles formed the inter-scholar cooperative network. Cluster#1 centered on Hashimoto N, Aoki T, Kataoka H, and Nozaki K, and most of them were Japanese scholars; Cluster#2 centered on Hashimoto T, Tada Y, Shimada K, and Kitazato K; and Cluster#3 centered on Meng Hui and Kolega J, and most of them were U.S. scholars.

### Keyword co-occurrence analysis

**Figure 5:**
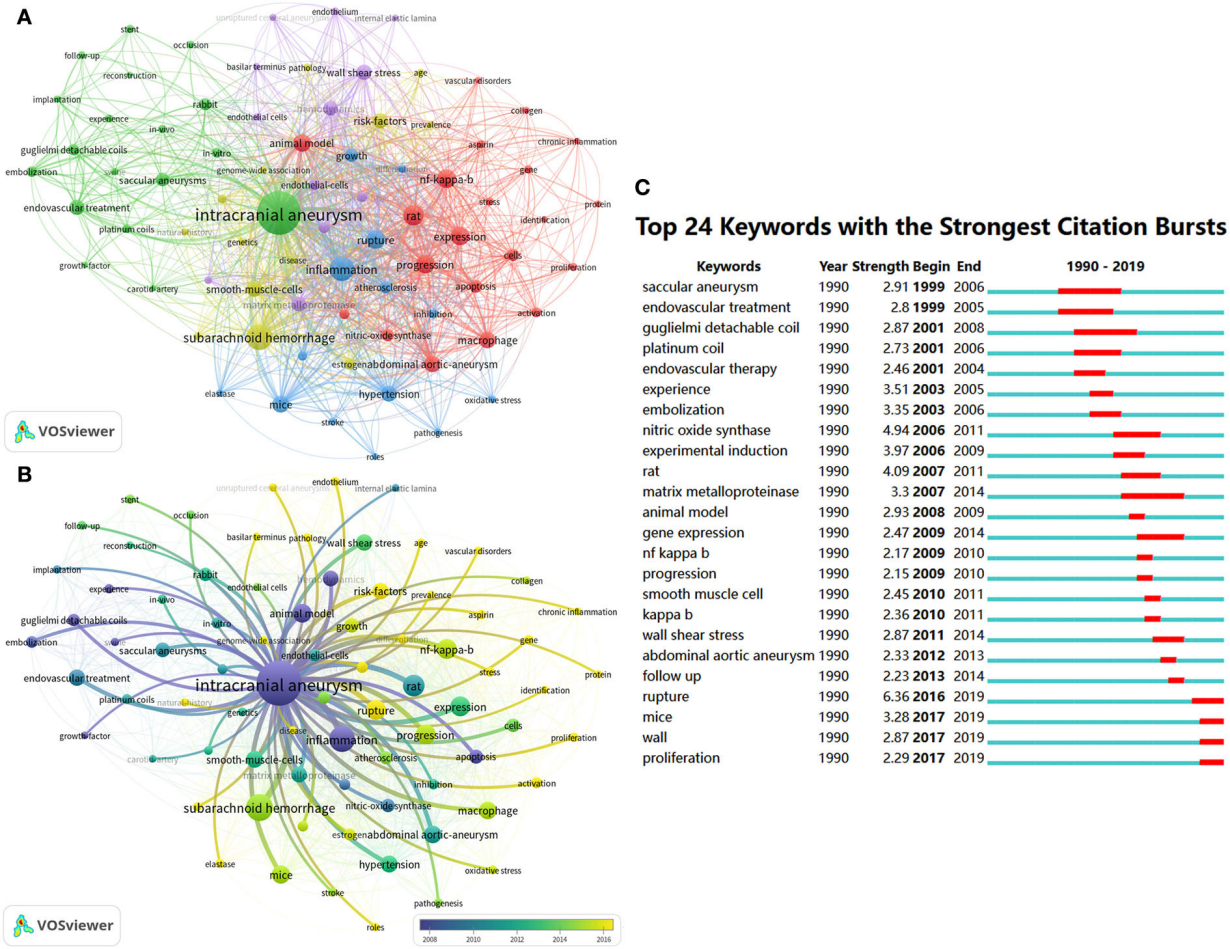
Keyword co-occurrence analysis related to IA anmial studies. The keyword co-occurrence network of animal IA studies **(A,B)**. Keywords clustered into five groups according to their color. “Rat-related IA research (cluster#red),” “endovascular treatment-related IA research (cluster#green),” “mice-related IA research (cluster#yellow),” “hemodynamics-related IA research (cluster#purple),” and “others (cluster#blue).” Large nodes represent keywords with high frequency; 24 keywords with the strongest citation bursts from 1990 to 2021 in CiteSpace are displayed. The red segment on the blue line indicates the burst duration **(C)**.

We analyzed keywords using VOSviewer and CiteSpace, with parameters set to default values. As shown in Figure 5A, 78 keywords that occurred more than five times in all articles were identified and grouped into five clusters. We renamed them as follows: “rat-related IA research (cluster#red),” “endovascular treatment-related IA research (cluster#green),” “mice-related IA research (cluster#yellow),” “hemodynamics-related IA research (cluster#purple),” and “others (cluster#blue).” In the cluster of “rat-related IA research,” the following keywords were mentioned frequently: “rat” (47 times), “expression” (40 times), “NF-kappa-B” (39 times), and “macrophage” (30 times). In the cluster of “endovascular treatment-related IA research,” relevant keywords included “intracranial aneurysm” (212 times), “endovascular treatment” (26 times), “coil” (23 times), and “rabbit” (18 times). In the cluster of “mice-related IA research,” primary keywords were “inflammation” (68 times), “rupture” (39 times), “mice” (33 times), and “hypertension” (29 times). In the cluster of “hemodynamics-related IA research,” primary keywords were “endothelial cell” (27 times), “wall shear stress” (26 times), “hemodynamics” (25 times), and “matrix metalloproteinase” (24 times). In the cluster of “others,” primary keywords were “subarachnoid hemorrhage” (74 times), “smooth muscle cells” (29 times), “risk factors” (27 times), and “estrogen” (13 times). Figures 5B,C display the evolution of keywords using the timeline view of VOSviewer and the keywords citation burst of CiteSpace. During the early stage of the IA-related animal research, “inflammation,” “animal model,” “hemodynamics,” and “apoptosis” were the primary focus. Then, focus shifted to studies related to “endovascular treatment,” “rabbit model,” and “rat model”. More recently, the keywords “subarachnoid hemorrhage,” “macrophage,” “rupture,” “mice,” “elastase,” “gene,” “protein,” “proliferation,” and “risk factors” received much attention.

### Co-cited references analysis

**Figure 6:**
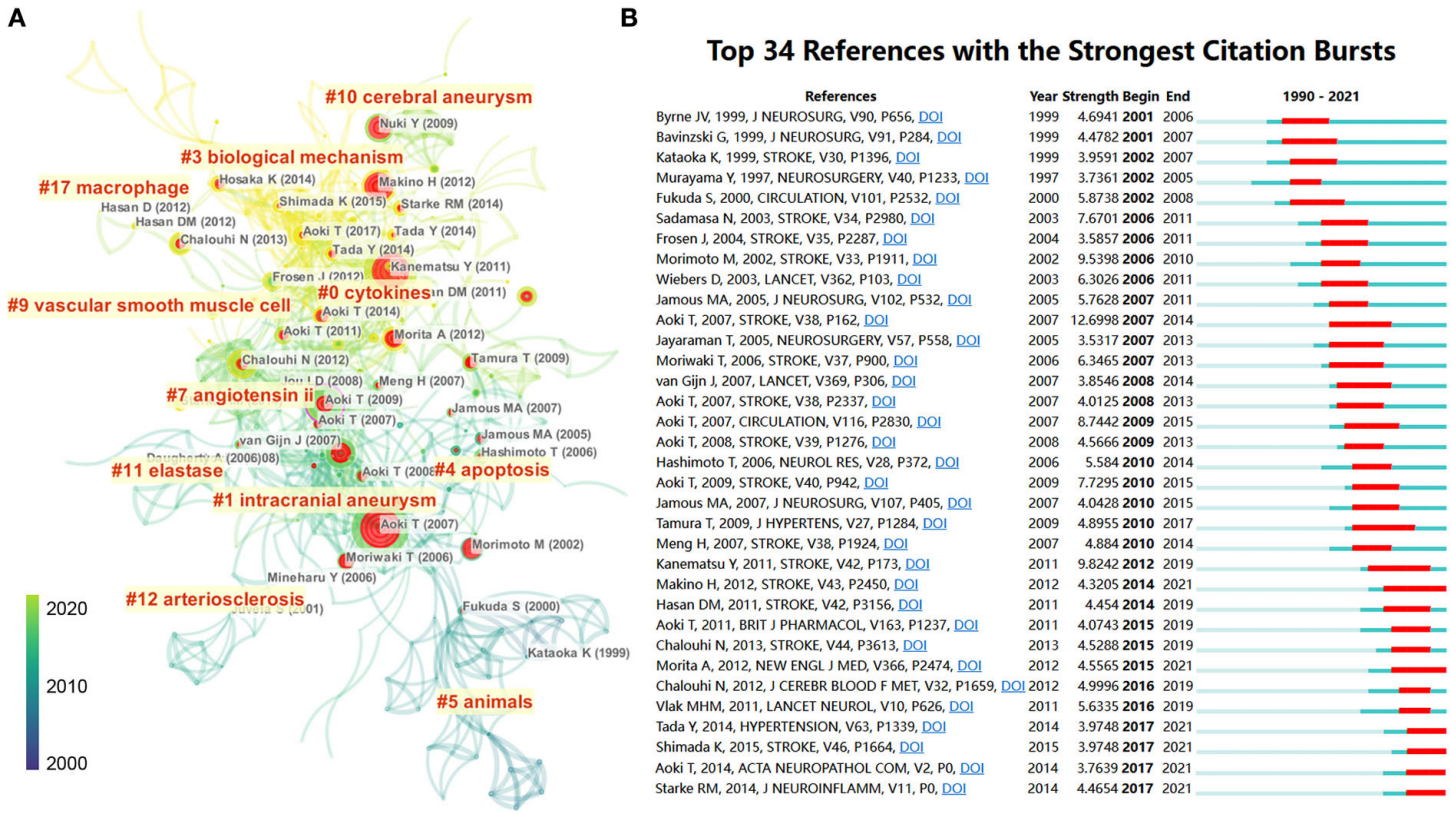
**(A)** Reference co-citation network identified by CiteSpace. The nodes and links are distinguished by colors, in which blue reflects an earlier co-citation relationship, and yellow represents a recent co-citation relationship. The size of nodes is positively associated with the citation number. The red nodes represent the publication has burst change, which may connect authors in different sub-domains in a given field. **(B)** The 34 most co-cited references with the strongest citation bursts represent important publications in different periods. The red bar indicates the burst duration. The burst strength indicates the importance of the research field.

Previous research forms the basis of scientific development. To display the scientific map and research evolution in this field, we used CiteSpace to identify articles with the strongest citation burst, which suggests that scholars in different periods focused on the topic. As shown in Figure 6A, researchers focused on different animal IA models with elastase or angiotensin II since an early period and shifted to exploring the biological mechanisms of IA. We visualized references with the strongest citation burst to identify key references in this field. In Figure 6B, 34 crucial publications in this field are listed on a timeline view map. The red bar indicates articles that received much attention during that time. Aoki T's article showed the highest citation burst (*n* = 12.69), where, by using a Sprague–Dawley (SD) rat IA model, the authors found that macrophage-derived MMP-2 and−9 could promote the progression of IA (29). Since 2015, nine publications have gained much attention from researchers: “Aoki T, 2011, BRIT J PHARMACOL, V163, P1237 (29),” “Chalouhi N, 2013, STROKE, V44, P3613 (40),” “Morita A, 2012, NEW ENGL J MED, V366, P2474 (41),” “Chalouhi N, 2012, J CEREBR BLOOD F MET, V32, P1659 (42),” “Vlak MHM, 2011, LANCET NEUROL, V10, P626 (2),” “Tada Y, 2014, HYPERTENSION, V63, P1339 (43),” “Shimada K, 2015, STROKE, V46, P1664 (44),” “Aoki T, 2014, ACTA NEUROPATHOL COM, V2, P0 (45),” and “Starke RM, 2014, J NEUROINFLAMM, V11, P0 (46).”

## Discussion

In this era of knowledge explosion, new researchers can quickly get insights into a particular research field through bibliometric analysis. Therefore, in this study, we retrieved IA publications using different animals, identified the main contributors, and visualized the evolution of research in this field over the past three decades.

### Publication trend

The number of IA-related animal studies has increased sharply since 2004. This significant increment is in line with the growth trend of animal studies, especially mice-related research, which surged in 2006. In 2002, Morimoto et al. (47) published one article in *Stroke*, entitled “*Mouse model of cerebral aneurysm-Experimental induction by renal hypertension and local hemodynamic changes*.” In this study, the authors experimentally induced saccular aneurysm at the right anterior cerebral artery–olfactory artery bifurcations in C57BL/6 male mice by ligating the left common carotid arteries and bilateral posterior renal arteries and feeding with a high-salt diet. This mice model allowed for genetic modifications and mechanistic probing of signaling pathways. The earliest article was published by Santos-Buch et al. (18) in 1976, which found that a higher rate of intracranial hemorrhage co-occurred with an iris aneurysm. However, this publication did not get a high number of citations. This may be due to the fact that, in 1978, Kido et al. (48) evidenced that cerebral microaneurysms induced in hypertensive rabbits primarily located near the basal ganglia and the cortex and not in the typical aneurysm locations (e.g., the internal carotid artery, the circle of Willis, and the middle cerebral artery).

### Journal

In terms of the journal, the three most prolific journals were *Stroke, Journal of Neurosurgery*, and *Neurosurgery*. Therefore, researchers should pay attention to the progress of these journals for their high-quality of IA-related animal studies. Notably, although there are only three publications in *Circulation* (IF = 39.9, 2021), two publications in the 10 most cited articles indicate that *Circulation* poses a high standard for IA-related animal studies.

### Country

The incidence of IA varies by region (2), and the level of interest in IA may vary in regions accordingly. The top three most productive countries were Japan (*n* = 97, 35.5%), the United States (*n* = 82, 30.0%), and China (*n* = 55, 20.1%). These countries have larger aging populations and higher levels of economic development and may pay special attention to basic research of IA. We believe these countries will continue to do so in future because scientific research can effectively guide national healthcare policies (49). The country co-authorship map made by VOSviewer provides information about the international cooperative network (17, 50). Japan and the United States have published the highest number of articles and secured a relatively important position in IA-related animal studies. The number of 10 most-cited articles and the nationality of the 10 most-cited authors also show Japanese and U.S. scholars' influence in this field. With the improvement of academic level and research funding, IA-related animal studies are launching in China. However, in the present study, we noticed that there are few influential articles and authors in China, and there is little cooperation between Chinese researchers and other countries. Several factors may explain the contradiction between China and the other two countries in regard to the quantity and quality of publications. First, compared with Japanese and U.S. scholars, Chinese scholars explored this domain relatively late. In Supplementary Figure S1, annual publications from China have shown a steady increment since 2011. In addition, our previous bibliometric analysis showed that, from 2012 to 2021, Chinese scholars were active in IA fields, especially after 2015 (13). In addition, we found that the percentage of publications in *Stroke, Journal of Neurosurgery*, and *Neurosurgery* (the highest citation journal) by authors from Japan and the United States was much higher than that from China (Supplementary Table S1).

### Author

Of the 10 most productive authors, eight were Japanese and two were Americans. Although China ranked third in terms of publications, Chinese scholars were absent from the 10 most prolific authors. In the timeline view of the author's cooperative network, Hashimoto N and Aoki T explored this field relatively early. Hashimoto N published a series of articles on experimentally induced cerebral aneurysms in rats by ligating the left common carotid artery and renal hypertension from the perspective of pathology, angiography, hemodynamics, and hypertension (19, 51, 52) from 1978 to 1993. Subsequently, the authors used the rat IA model to explore the functions of apoptotic smooth muscle cells (53) and nitric oxide synthase (30). In 2002, Hashimoto N also produced the mice IA model through ligation of the left common carotid artery and renal hypertension (47). In 2006, Aoki T and Hashimoto N cooperated and published the first publication using both rat and mice IA models, entitled “Impaired progression of cerebral aneurysms in interleukin-1 beta-deficient mice (54).” Thereafter, they explored the functions of several genes in the development of IA, including macrophage-derived matrix metalloproteinase 2 and 9 (29), tissue inhibitor of matrix metalloproteinase (55), NF-kappa (34), apolipoprotein E (56), cysteine cathepsins (B, K, and S) (57), monocyte chemoattractant protein-1 (58), and Toll-like receptor 4 (59). Apart from investigating the functions of specific genes, they also evidenced several drugs that could suppress the progression of IA *in vivo*, including simvastatin (60), pitavastatin (61), and nifedipine (62).

### Research trend and frontier topics

#### Aneurysm model development

In the early stage, scholars focused on constructing various animal IA models; exploring the relationship between several phenotypes, like apoptosis, inflammation, and macrophage; and evaluating endovascular coil applications. For example, in 1978, Hashimoto N had successfully induced saccular cerebral aneurysms in rats, and in 2002, they induced aneurysmal change in mice. In 2007, Meng Hui successfully generated aneurysmal change in the carotid artery using dogs (36). Subsequently, to simulate the intracranial microenvironment, they induced a basilar apex aneurysm by ligating the bilateral carotid artery in rabbits (63). The three aforementioned classic animal models laid a solid foundation for subsequent research. However, they also have significant drawbacks. It often takes up to 3 months to observe the lower rate of minimal aneurysmal remodeling in these IA models. In 2009, Nuki et al. induced an aneurysm in the circle of Willis of mice with an elastase (35 mU) injection into the basal cistern and sustained infusion of angiotensin II (500 or 1,000 ng/kg/min) to produce hypertension. This model significantly shortened the time (1 month) to make an obvious human-like saccular intracranial aneurysm with a 77% success rate (64). Afterward, in 2012, Makino et al. also modified this model using mice with elastase stereotactic injection and hypertension (unilateral nephrectomy, DOCA-salt pellet implantation, and 1% NaCl in the diet). It enabled 50–60% of created IAs to rupture within 2 weeks. However, the fast delivery of elastase does not occur during human IA progression. It may mask the actual chronic biological mechanisms behind the IA rupture. Future research may induce the arterial collagen degradation slowly and modestly to better simulate the actual growth and rupture phenomenon in IA.

#### Aneurysm mechanism exploration

Recently, the treatment of IA shifted from open surgical clipping to endovascular therapies due to the advancements in neuroendovascular devices. The animal model can be ideally used to test the efficacy of coil embolization and stent implantation. Accordingly, as shown in Figure 5B, the rabbit model and the swine model are often used in testing of novel endovascular therapies before clinical application. For example, Bearat et al. evaluated the cytotoxicity, viscoelastic behavior, and degradation of dual-gelling poly (N-isopropylacrylamide)-based polymer systems for IA embolization using the swine IA model (65). Moftakhar et al. tested two versions of an aneurysm intra-saccular occlusion device to address the higher rate of aneurysm recurrence using the swine and canine models (66). Adibi et al. reported that an adjuvant mesenchymal stem/stromal cell (MSC) infusion combined with coil embolization will improve histological healing in a rabbit elastase aneurysm model. This may accelerate the aneurysm repair process and minimize adverse complications (67). The most critical drawback of coiling is a higher aneurysm recurrence rate, particularly in wide-necked or large aneurysms. Recently, stent-assisted coiling or flow diversion was largely used to treat wide-necked or large aneurysms. However, the prolonged use of antiplatelet agents may result in a higher rate of bleeding complications. Arai et al. developed a stent that can control the release of basic fibroblast growth factor (bFGF) and biodegradable poly(D,L-lactide-co-glycolide) (PLGA) argatroban to accelerate the recovery of an aneurysm in the elastase rabbit model. The authors found that the occurrence rate of an in-stent thrombus is low in drug-coated stents, which may be used in the clinic in future (68). In addition to developing new devices, animal models are more commonly used to study the biological mechanics of aneurysm formation. Topics such as inflammation, oxidative stress, rupture, NF-κB, macrophage, endothelium, subarachnoid hemorrhage, smooth muscle cells, gene, protein, proliferation, and risk factors have received much attention since 2015 (yellow-green or yellow in Figure 5B). Indeed, a recent study published in *Circulation* demonstrated that endothelial SOX17-deficient mice are prone to induce IA under hypertensive conditions, and targeting SOX17 may accelerate the drug treatment for IA (69). Another study showed that the incidence of aneurysms and subarachnoid hemorrhage was significantly lower in myeloperoxidase knockout mice than in wild-type mice, suggesting that reducing the level of systemic oxidative stress may be effective in preventing the initiation and rupture of IA (70). The mechanisms of several evidenced risk factors have also been explored in recent years. Starke et al. reported that cigarette smoking could initiate oxidative stress-induced vascular smooth muscle cell phenotypic switching and promote IA progression using the mice IA model (71). Yanagisawa et al. found that the rate of aneurysm rupture and the presence of unruptured aneurysms significantly differed among different strains or sex of mice (72). In addition, scholars also investigated the mechanisms of various cytokines, inflammation factors, and inflammation cells in the development of IA, including interleukin-6 (73), monocyte chemotactic protein-1–interleukin-6–osteopontin pathway (74), toll-like receptor 4 (TLR4) (75), tumor necrosis factor alpha (45, 46), lymphocytes (76), mast cells (77), M1 macrophages (78). However, detailed mechanisms of IA are largely unknown. Exploring the molecular mechanisms of IA using mouse models of IA and thus developing targeted drugs remain important research topics now and will likely be in future.

### Limitation

First, the search term used in this article is “intracranial aneurysm,” “cerebral aneurysm,” or “brain aneurysm” in the title to accurately sort out articles related to animal IA research. Because the animal model of SAH is significantly different from the animal model of IA, and scholars studying SAH often focus on the pathophysiologic changes of brain tissue besides the cerebrovascular system, we excluded publications that mainly discussed SAH. So, our search terms inevitably removed some publications focusing on aSAH. Second, the publications of IA-related animal studies in the current study were extracted from the WOS. Although the WOS is one of the most recognized authoritative databases, other databases such as PubMed, Scopus, and Google Scholar are also recognized by scientists. Third, citation counts were used as an important indicator to evaluate the quality, and the influence of publications is still controversial because the journal impact factor, H-index, m-quotient, and authorship value also provide a good reference value (8). Fourth, we included only English publications in this survey. Several studies related to animal IA models in non-English languages, such as Chinese and Japanese, may be ignored. Lastly, articles were only included till July 2021, which means that the data for 2021 in this bibliometric analysis is incomplete. However, we believe that the low citation frequency of recent publications will not affect the main conclusions. We believe this bibliometric analysis will provide a beneficial reference to the main points, as well as the future trends, of IA-related animal research, not only for researchers who have already been working in this field but also for new researchers preparing to become active members of this field.

## Conclusion

In summary, the current research provides a beneficial reference to the classical documents and the future trends of IA-related animal research. Japan and the United States have made the greatest contributions to this field, in terms of both researchers and institutions. China ranked third in that line; however, it still lacks high-quality articles and influential institutions. High-quality articles can be found in *Stroke* and *Journal of Neurosurgery, Neurosurgery*, and *Circulation*. Meng hui, Hashimoto N, Hashimoto T, and Aoki Tomorito are ideal candidates for academic cooperation. Rats and mice IA models are primarily used for molecular mechanism research. The rabbit and swine models are mainly used for studying hemodynamics and evaluating the applicability of interventional materials. Using mice and rat models of IA to investigate the molecular mechanisms of IA is a significant topic in current research and is likely to continue in future.

## Data availability statement

The original contributions presented in the study are included in the article/Supplementary material, further inquiries can be directed to the corresponding author.

## Author contributions

JC and JL designed this study and drafted the manuscript. JC and XL involved in study design, obtained data, contributed to interpretation, and helped to draft the manuscript. QZ provided the theoretical frameworks and performed much of the editing of the manuscript. ZC, CZ, and SL helped a lot in the revision process, collecting data, organizing literature, and redo the figures and tables. All authors contributed to the article and approved the submitted version.

## Funding

This study was supported by the Hunan Science and Technology Innovation Platform and Talent Plan (Grant: 2017TP1004).

## Conflict of interest

The authors declare that the research was conducted in the absence of any commercial or financial relationships that could be construed as a potential conflict of interest.

## Publisher's note

All claims expressed in this article are solely those of the authors and do not necessarily represent those of their affiliated organizations, or those of the publisher, the editors and the reviewers. Any product that may be evaluated in this article, or claim that may be made by its manufacturer, is not guaranteed or endorsed by the publisher.

## Supplementary material

Supplementary Figure 1 Publication trend of Japan, the USA, and China.
