# Supplementary figures and images for: Animal model contributes to the development of intracranial aneurysm: A bibliometric analysis
Source: Front Vet Sci. 2022 Nov 18;9:1027453. doi: 10.3389/fvets.2022.1027453 (PMC9716216; doi:10.3389/fvets.2022.1027453)

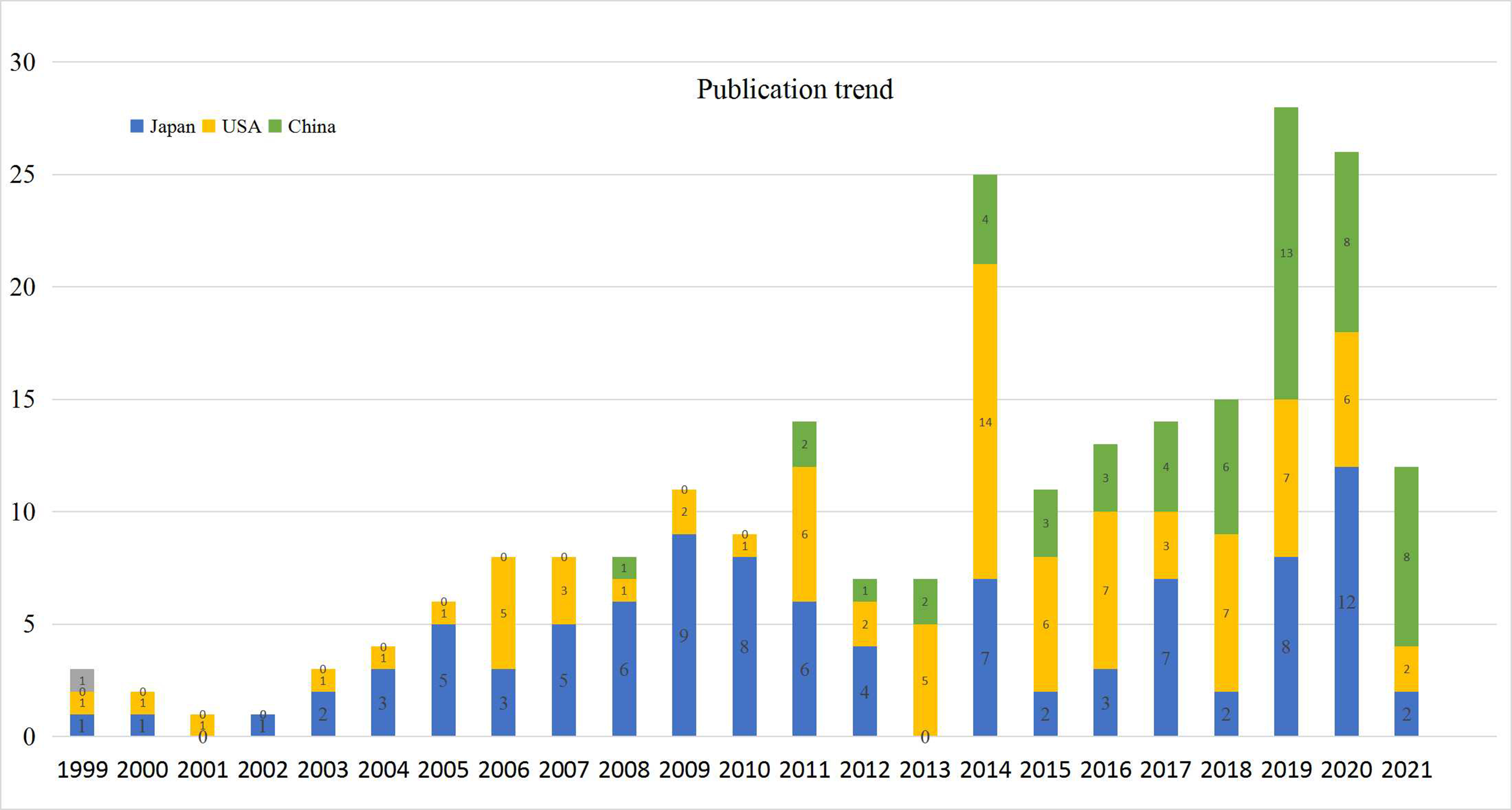

Supplement: Supplementary Figure 1 — Publication trend of Japan, the USA, and China. [file Image_1.JPEG]
