# Supplementary material for: Animal model contributes to the development of intracranial aneurysm: A bibliometric analysis
Source: Front Vet Sci. 2022 Nov 18;9:1027453. doi: 10.3389/fvets.2022.1027453 (PMC9716216; doi:10.3389/fvets.2022.1027453)
Supplement: Supplementary file 2 [file Table_1.docx]

Table S1. The top 10 journal distribution of three prolific countries

| Journal | Publications |
| --- | --- |
| Japan | |
| Stroke | 15 |
| Journal of Neurosurgery | 13 |
| Neurosurgery | 6 |
| Neurologia Medico Chirurgica | 5 |
| Hypertension | 3 |
| International Journal of Molecular Medicine | 3 |
| Journal of Neuroinflammation | 3 |
| Journal of The American Heart Association | 3 |
| Acta Neuropathologica Communications | 2 |
| American Journal of Neuroradiology | 2 |
| USA | |
| Stroke | 16 |
| Hypertension | 8 |
| American Journal of Neuroradiology | 6 |
| Journal of Neurointerventional Surgery | 6 |
| Neurosurgery | 5 |
| Journal of Neuroinflammation | 4 |
| Journal of Cerebral Blood Flow and Metabolism | 3 |
| Journal of Neurosurgery | 3 |
| Plos One | 3 |
| Translational Stroke Research | 3 |
| China | |
| International Journal of Clinical and Experimental Medicine | 3 |
| Biochemical and Biophysical Research Communications | 2 |
| Cellular and Molecular Biology | 2 |
| Cns Neuroscience Therapeutics | 2 |
| Journal of Clinical Neuroscience | 2 |
| Journal of Neuroinflammation | 2 |
| Molecular Medicine Reports | 2 |
| Nanoscale Research Letters | 2 |
| Neuroradiology | 2 |
| World Neurosurgery | 2 |
